# Lymphoid-biased hematopoietic stem cells and myeloid-biased hematopoietic progenitor cells have radioprotection activity

**DOI:** 10.1097/BS9.0000000000000089

**Published:** 2021-08-19

**Authors:** Shanshan Zhang, Aled O’Neill, Miner Xie, Peng Wu, Xiaofang Wang, Haitao Bai, Fang Dong, Jinhong Wang, Qingyun Zhang, Toshio Suda, Hideo Ema

**Affiliations:** a State Key Laboratory of Experimental Hematology, National Clinical Research Center for Hematological Disorders, Institute of Hematology and Blood Diseases Hospital, Chinese Academy of Medical Sciences and Peking Union Medical College, Tianjin, China; b Department of Cell Differentiation, Sakaguchi Laboratories of Developmental Biology, Keio University School of Medicine, Tokyo, Japan

**Keywords:** Hematopoietic progenitor cells (HPCs), Hematopoietic stem cells (HSCs), Lymphoid-biased hematopoietic stem cells, Myeloid-biased hematopoietic progenitor cells, lymphoid-primed multipotent progenitors (LMPPs), Myeloid-biased hematopoietic stem cells, Radioprotection

## Abstract

Radioprotection was previously considered as a function of hematopoietic stem cells (HSCs). However, recent studies have reported its activity in hematopoietic progenitor cells (HPCs). To address this issue, we compared the radioprotection activity in 2 subsets of HSCs (nHSC1 and 2 populations) and 4 subsets of HPCs (nHPC1–4 populations) of the mouse bone marrow, in relation to their in vitro and in vivo colony-forming activity. Significant radioprotection activity was detected in the nHSC2 population enriched in lymphoid-biased HSCs. Moderate radioprotection activity was detected in nHPC1 and 2 populations enriched in myeloid-biased HPCs. Low radioprotection activity was detected in the nHSC1 enriched in myeloid-biased HSCs. No radioprotection activity was detected in the nHPC3 and 4 populations that included MPP4 (LMPP). Single-cell colony assay combined with flow cytometry analysis showed that the nHSC1, nHSC2, nHPC1, and nHPC2 populations had the neutrophils/macrophages/erythroblasts/megakaryocytes (nmEMk) differentiation potential whereas the nHPC3 and 4 populations had only the nm differentiation potential. Varying day 12 spleen colony-forming units (day 12 CFU-S) were detected in the nHSC1, nHSC2, and nHPC1–3 populations, but very few in the nHPC4 population. These data suggested that nmEMk differentiation potential and day 12 CFU-S activity are partially associated with radioprotection activity. Reconstitution analysis showed that sufficient myeloid reconstitution around 12 to 14 days after transplantation was critical for radioprotection. This study implied that radioprotection is specific to neither HSC nor HPC populations, and that lymphoid-biased HSCs and myeloid-biased HPCs as populations play a major role in radioprotection.

## 1 INTRODUCTION

Radioprotection can be defined as the condition of being protected from the lethal effect of irradiation. For instance, it is observed when lethally irradiated mice are rescued by bone marrow transplantation. Thus, bone marrow cells have radioprotection activity. Radioprotection has been considered as one of the functions of hematopoietic stem cells (HSCs).^1–3^ However, as the purification of HSCs and hematopoietic progenitor cell (HPCs) has progressed, HPCs have been considered to be primarily responsible for radioprotection.^4–6^ Since then, less attention has been paid to the radioprotection activity in HSCs. Recently, we have suggested that HSCs are enforced to differentiate at an early time after transplantation.^7^ This has raised the possibility that HSCs also contribute to radioprotection. In this study, we asked whether both HSCs and HPCs have radioprotection activity.

The number of day 12 CFU-S and radioprotection activity in the bone marrow of W mutant mice were found to be significantly reduced.^8^ Day 12 spleen colonies appeared to develop depending on the SCF/c-Kit signal.^9^ Day 12 CFU-S activity and radioprotection were suggested to be associated.^10,11^

We have recently characterized 2 HSC and 4 HPC populations^12^: CD201^+^CD150^+^CD48^−^CD41^−^CD34^−^Kit^+^Sca-1^+^Lin^−^ cells (nHSC1), CD201^+^CD150^−^CD48^−^CD41^−^CD34^−^Kit^+^Sca-1^+^Lin^−^ cells (nHSC2), CD201^+^CD150^+^CD48^−^CD41^+^CD34^−^Kit^+^Sca-1^+^Lin^−^ cells (nHPC1), CD150^+^Flt3^−^CD34^+^KSL cells (nHPC2), CD150^−^Flt3^−^CD34^+^KSL cells (nHPC3), and CD150^−^Flt3^+^CD34^+^KSL (nHPC4) cells. In this study, we compared the radioprotection activity, in vitro colony-forming activity, and in vivo CFU-S activity among these populations. Varying degrees of these activities were detected among HSCs and HPCs. Neither high in vitro nor in vivo colony-forming activity was a sufficient condition of radioprotection. This study suggested that appropriate timing, degree, and context of myelopoiesis are required for radioprotection.

## 2 RESULTS

### 2.1 Single-cell colony assays

**Figure 1:**
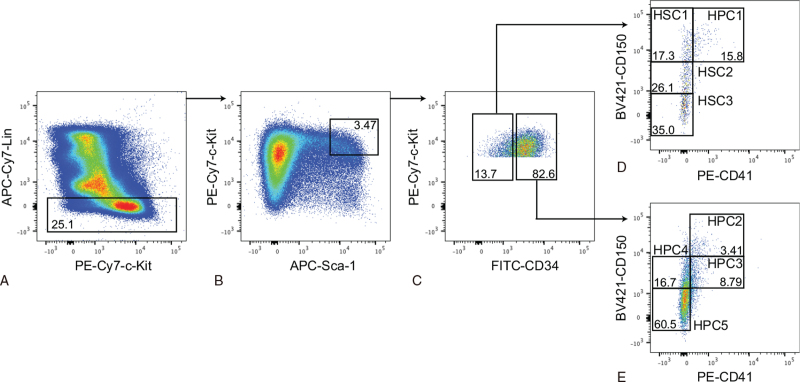
Flow cytometry sorting gates. Bone marrow cells from B6 mice were stained with antibodies. (**A**) Lineage-negative cells were gated. (**B**) c-Kit^+^Sca-1^+^lineage-negative (KSL) cells were gated. (**C**) Cells were then divided into CD34^−^ and CD34^+^ fractions. (**D**) The CD34^−^ fraction was further divided into 4 populations, the HSC1 (CD150^High^CD41^−^), HSC2 (CD150^Mid^CD41^−^), HSC3 (CD150^Low^CD41^−^), and HPC1 (CD150^High^CD41^+^) populations. (**E**) The CD34^+^ fraction was also divided into 4 populations, the HPC2 (CD150^High^CD41^+^), HPC3 (CD150^Mid^CD41^+^), HPC4 (CD150^Mid^CD41^−^), and HPC5 (CD150^Low^CD41^−^) populations. HPCs = hematopoietic progenitor cells, HSCs = hematopoietic stem cells.

Kit^+^Sca-1^+^Lin^−^ (KSL) cells in mouse bone marrow are significantly enriched in HSC and HPC activities.^2^ CD34 expression levels can be used to separate HSCs from HPCs.^4^ Furthermore, CD150 expression level can be used to separate lymphoid-biased HSCs from myeloid-biased HSCs.^13,15–17^ Accordingly, the HSC1, HSC2, HSC3, HPC1, HPC2, HPC3, HPC4, and HPC5 populations were defined as shown in Figure 1.

Single-cell colony assays were used to compare the proliferation and differentiation potentials of the HSC1, 2, and 3 populations and the HPC1, 2, 3, 4, and 5 populations, which accounted for 2.2 ± 1.5%, 5.0 ± 1.8%, 3.6 ± 2.3%, 3.4 ± 1.6%, 3.8 ± 1.6%, 10.5 ± 4.5%, 34.7 ± 12.7%, and 31.2 ± 12.5% of KSL cells, respectively (mean ± SD, *n* = 20). After photographs were taken, individual colonies were stained with antibodies and analyzed by flow cytometry. After 7 and 14 days of culture, the numbers of live and dead cells and the proportions of cells of each lineage per colony were determined.

**Figure 2:**
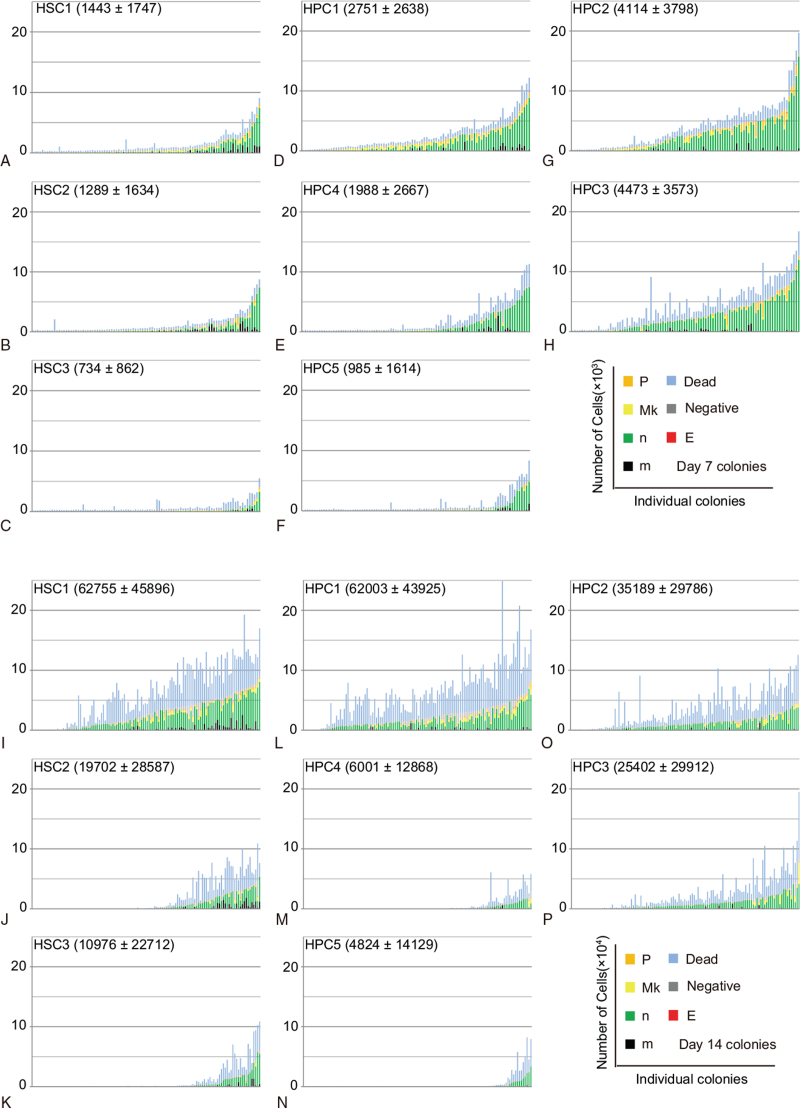
Single-cell colony formation after 7 and 14 days of culture. **(A-H)** Colonies at day 7 of culture were organized in ascending order according to the number of living cells. The top 96 wells with the greatest number of cells produced by single cells from the **(A)** HSC1, **(B)** HSC2, **(C)** HSC3, **(D)** HPC1, **(E)** HPC4, **(F)** HPC5, **(G)** HPC2, and **(H)** HPC3 populations are shown. The number of cells per well are shown as mean ± SD (n = 96). Each column comprised m, n, Mk, P, E, lineage-negative cells, and dead cells. Dead cells and lineage marker-negative cells (negative) are also included (blue and gray columns, respectively). **(I-P)** Colonies at day 14 were organized in ascending order according to the number of living cells. The top 120 wells with the greatest number of cells produced by single cells from the **(I)** HSC1, **(J)** HSC2, **(K)** HSC3, **(L)** HPC1, **(M)** HPC4, **(N)** HPC5, **(O)** HPC2, and **(P)** HPC3 populations are shown. The number of cells per well are shown as mean ± SD (n = 120). Each column comprised m, n, Mk, P, E, lineage-negative cells, and dead cells. Dead cells and lineage marker-negative cells (negative) are also included (blue and gray columns, respectively). HPCs = hematopoietic progenitor cells, HSCs = hematopoietic stem cells.

In total, over 2000 individual colonies were analyzed. Cells were stained with antibodies against individual colonies, and neutrophils (n), macrophages (m), megakaryocytes (Mk), erythrocytes (E), and Mk/E progenitors (P) were identified by flow cytometry. Fluorospheres were simultaneously used to count the cells in each colony. The numbers of n, m, Mk, E, and P produced by single cells from 8 populations after 7 and 14 days of culture are shown in Figure 2. Notably, the HPC1–3 populations produced colonies with a significantly greater number of cells per colony after 7 days of culture than the other populations of cells did, but there was no significant difference in the number of cells per colony among the HPC1–3 populations. After 14 days of culture, HSC1 and HPC1 colonies contained a significantly greater number of cells per colony than the colonies of other populations of cells, but there was no significant difference in the number of cells per colony between the HSC1 and HPC1 populations. The number of cells per colony among HSC2, HSC3, HPC4, and HPC5 was also increasing, but at a slower rate.

**Figure 3:**
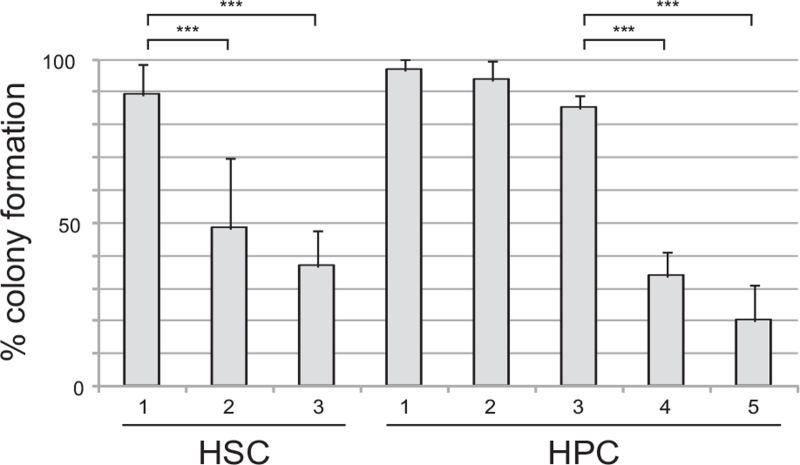
Colony-forming efficiencies of the HSC1–3 and HPC1–5 populations. The percentages of cells in the HSC1, HSC2, HSC3, HPC1, HPC2, HPC3, HPC4, and HPC5 populations that formed colonies after 14 days of culture are shown. The colony-forming efficiency of the HSC1 population was significantly greater than those of the HSC2 and HSC3 populations (*P* < .0001). The colony-forming efficiencies of the HPC1, HPC2, and HPC3 populations were significantly greater than those of the HPC4 and HPC5 populations (*P* < .0001). Data are shown as mean ± SD (n = 10 for the HSC1–3 and HPC1 populations; n = 5 for the HPC2–5 populations). HPCs = hematopoietic progenitor cells, HSCs = hematopoietic stem cells.

Figure 3 summarizes the colony-forming efficiency of each group after 14 days of culture. Over 80% of cells from the HSC1 and HPC1, 2, and 3 populations gave rise to colonies. The colony-forming efficiency of the HSC1 population appeared to be significantly greater than those of the HSC2 and 3 populations. Similarly, the colony-forming efficiencies of the HPC1, 2, and 3 populations were significantly greater than those of the HPC4 and 5 populations.

### 2.2 The similar colony-forming potentials between the HSC1 and HPC1 or HPC4 and HPC5 populations

**Figure 4:**
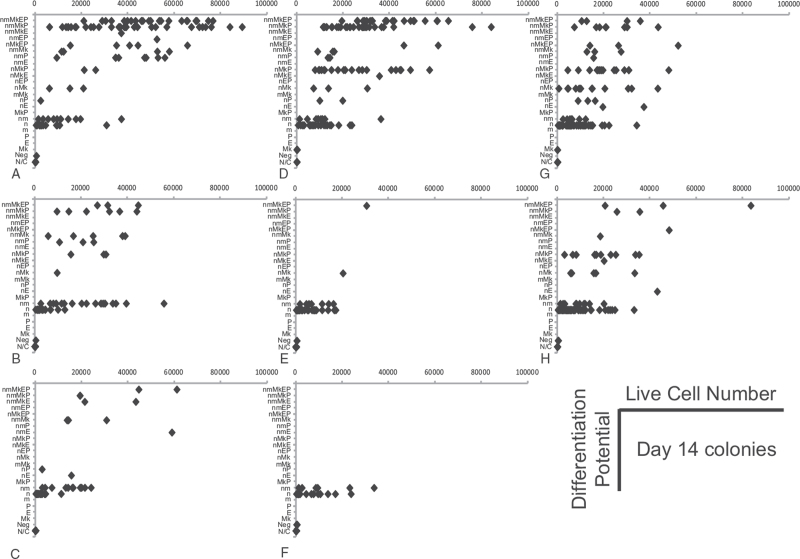
Differentiation potentials and number of cells per colony after 14 days of culture. The myeloid lineage differentiation potential and a number of live cells detected in individual colonies after 14 days of culture are shown for the (**A)** HSC1, **(B)** HSC2, **(C**) HSC3, **(D**) HPC1, (**E**) HPC4, **(F**) HPC5, **(G**) HPC2, and **(H**) HPC3 populations. Neutrophils (n), monocytes (m), megakaryocytes (Mk), erythroblasts (E), and progenitors (Ps). Negative colonies contained live cells without lineage markers (Neg). No colony was found (N/C). HPCs = hematopoietic progenitor cells, HSCs = hematopoietic stem cells.

The HSC1 and HPC1 populations showed a variety of differentiation potentials during the in vitro culture. Figure 4 summarizes the relationship between differentiation potential and cell number per colony on day 14 of culture. The cell distribution patterns of the HSC1 and HPC1 populations in these graphs were remarkably similar. A total of 40% to 50% of cells in the HSC1 and HPC1 populations gave rise to nmEMk and nmMk (multilineage) colonies, which were significantly larger than nm colonies from these 2 populations. While the multilineage colonies were not detected on day 7 but had become detectable by day 14, some of them increased in size by day 14 (Fig. 2). In the case of the HSC1 population, the nm and Mk lineages, but not the E lineage, were detectable beginning at day 7, suggesting that these lineages arise during the early stages of HSC differentiation. Of note is that multilineage colonies were formed by HSC1–3 and HPC1–3 cells.

In contrast to these results, a skewed distribution of differentiation potentials among the HPC4/5 populations was observed. Almost all cells in the HPC4/5 populations gave rise to nm colonies (Fig. 4). After 7 days of culture, only a small number of nm colonies had been produced by HSC3 cells, although many nm colonies had been formed by cells in the other populations, including the HSC1 population. These data suggested that cells in the HSC3 population with nm differentiation potential are more immature than the cells of other populations with nm differentiation potential.

### 2.3 Revised HSC and HPC populations

To study B and T cell differentiation potentials, we next performed a transplantation assay. To see more significant differences among populations, we redefined the HSC and HPC populations with gaps between the populations. We additionally used CD48 and CD201 to increase the purity of the cells. We also replaced CD41 with Flt-3 for the detection of HPC populations. The new HSC1 and 2 populations were designated the nHSC1/2 populations, while the new HPC1–4 populations were designated the nHPC1–4 populations. Their sorting gates and the relationship between the previously defined and redefined populations are shown in Supplemental Fig. 1 and Supplemental Table 1. CD48^+^ cells and CD201^−^ cells were excluded from the HSC1, HSC3, and HPC1 populations to define the nHSC1, nHSC2, and nHPC1 populations, respectively. The nHPC2 population included the HPC2 population and parts of the HPC3 and 4 populations. The nHPC3 and 4 populations overlapped the HPC5 population (see Supplemental Table 1 for their relationship). The nHSC1, nHSC2, nHPC1, nHPC2, nHPC3, and nHPC4 populations accounted for 4.9 ± 1.5%, 15.3 ± 4.0%, 1.2 ± 0.8%, 6.1 ± 1.5%, 21.5 ± 3.2%, and 53.7 ± 3.6% of KSL cells, respectively (mean ± SD, n = 10).

### 2.4 Survival curves of lethally irradiated mice after transplantation

**Figure 5:**
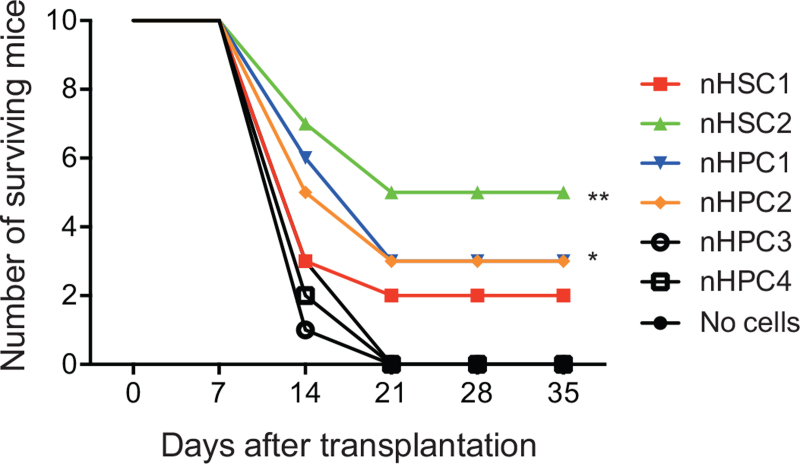
Survival curves of lethally irradiated mice after transplantation. One hundred cells each were sorted from the nHSC1, nHSC2, nHPC1, nHPC2, nHPC3, and nHPC4 populations of B6-CD45.1 mice and transplanted into 10 lethally irradiated B6-CD45.2 mice. ∗*P* < .05; ∗∗*P* < .01 (Log-rank test). HPCs = hematopoietic progenitor cells, HSCs = hematopoietic stem cells.

Hundred cells each from the nHSC1, nHSC2, nHPC1, nHPC2, nHPC3, and nHPC4 populations were transplanted into 10 lethally irradiated mice. Mice transplanted with no cells, nHPC3 cells, and nHPC4 cells similarly died by day 21 after transplantation (Fig. 5). In contrast, mice transplanted with nHSC2, nHPC1, and nHPC2 cells survived significantly longer than those 3 groups of mice. A small number of mice transplanted with nHSC1 also survived. These data showed the radioprotection activity was detectable in a small number of cells from nHSC1/2 and nHPC1/2. Its activity appeared to be greater in nHSC2 and nHPC1/2.

### 2.5 CFU-S activity and early reconstitution

**Figure 6:**
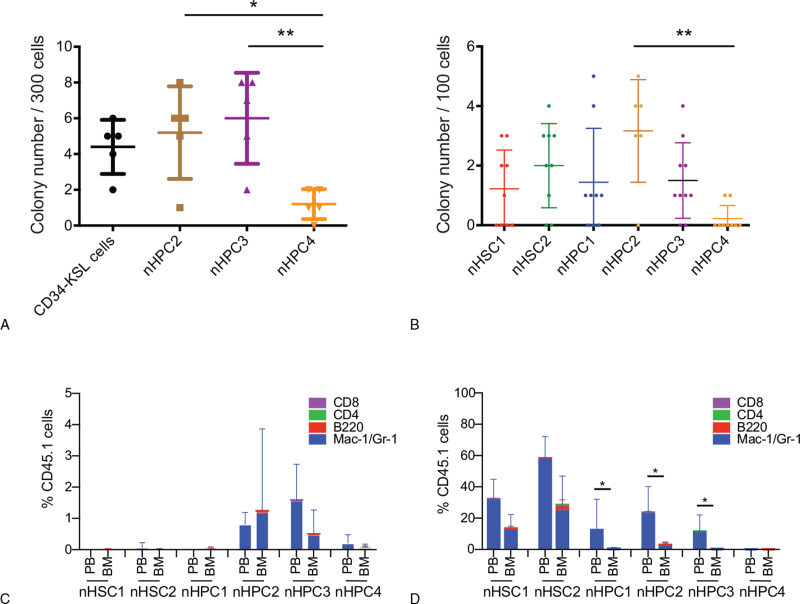
CFU-S and early reconstitution potential among the nHSC1, 2 and nHPC1–4 populations. **(A, B)** The number of day 12 spleen colonies is shown. ∗*P* < .05; ∗∗*P* < .01 (ANOVA). **(A)** Three-hundred CD34^−^KSL cells, nHPC2, nHPC3, and nHPC4 cells from B6-CD45.1 mice were transplanted into 5 lethally irradiated B6-CD45.2 mice. **(B)** One hundred nHSC1, nHSC2, nHPC1, nHPC2, nHPC3, and nHPC4 cells from B6-CD45.1 mice were transplanted into 10 lethally irradiated B6-CD45.2 mice. **(C, D)** One hundred nHSC1, nHSC2, nHPC1, nHPC2, nHPC3 and nHPC4 cells from B6-CD45.1 mice were transplanted into 5 lethally irradiated B6-CD45.2 mice. **(C)** PB and BM analysis on day 7 after transplantation. **(D)** PB and BM analysis on day 12 after transplantation. Myeloid reconstitution in PB was significantly greater than that on BM (∗*P* < .05). ANOVA = analysis of variance, BM = bone marrow, CFU-S = spleen colony-forming units, HPCs = hematopoietic progenitor cells, HSCs = hematopoietic stem cells, PB = peripheral blood.

We performed a CFU-S assay with 300 cells each from the nHPC2, nHPC3, nHPC4, and CD34^−^KSL populations. nHPC2/3 cells formed significantly more day 12 colonies than did nHPC4 cells (Fig. 6A), consistent with previous studies.^11^ Notably, in support of previous studies,^18^ we observed a small but significant number of day 12 CFU-S in CD34^−^ KSL cells.

Next, 100 cells each from the nHSC1, nHSC2, nHPC1, nHPC2, nHPC3, and nHPC4 populations were transplanted into lethally irradiated mice. Day 12 CFU-S were detected in all populations, consistent with a previous study.^18^ The number of day 12 CFU-S in the nHPC2 population was significantly greater than that in the nHPC4 population (Fig. 6B), consistent with a previous study.^11^ In addition, there was no significant difference in colony number among the other groups. We also performed a day 7 CFU-S assay with 100 cells from each population. We did not detect any day 7 colonies among these populations (data not shown).

We also analyzed the peripheral blood (PB) and bone marrow (BM) on days 7 and 12 after the transplantation of 100 cells each from the nHSC1, nHSC2, nHPC1, nHPC2, nHPC3, and nHPC4 populations (Fig. 6C and D). Low levels of myeloid reconstitution were detected in the PB and BM on day 7 after transplantation with nHSC2, 3, and 4 cells. However, no early reconstitution was detected in the PB and BM after transplantation with nHSC1, nHSC2, and nHPC1 cells (Fig. 6C). Myeloid reconstitution was detected in the PB and BM on day 12 after the transplantation with nHSC1/2 cells. Myeloid reconstitution in the PB was greater than that in the BM on day 12 after the transplantation of cells in nHPC1, 2, and 3 cells (Fig. 6D). These data showed that nHPC2, 3, and 4 cells reconstituted myeloid lineage at least 5 days earlier than nHSC1, nHSC2, and nHPC1 cells. These data suggested that day 12 but not day 7 myelopoiesis may be in part associated with radioprotection.

### 2.6 Reconstitution of surviving mice

**Figure 7:**
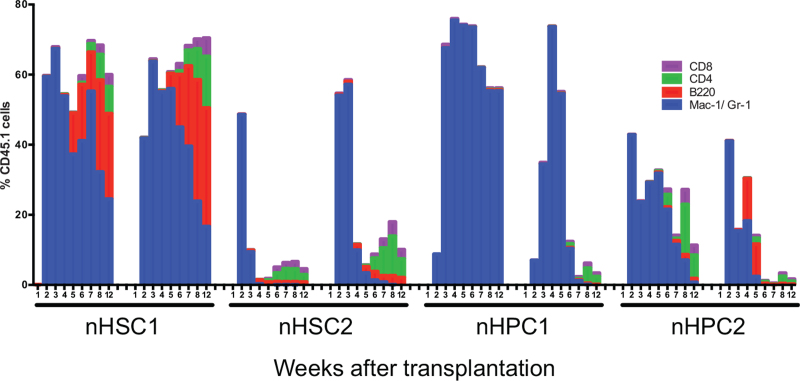
Multilineage reconstitution in survived mice. One hundred cells each were sorted from the nHSC1, nHSC2, nHPC1, nHPC2, nHPC3, and nHPC4 populations of B6-CD45.1 mice and then transplanted into 10 lethally irradiated B6-CD45.2 mice without competitor cells. Data of the representative 2 mice which survived 12 weeks after transplantation are shown. All recipients of nHSC3 and nHPC4 cells died. The percentages of chimerism comprising granulocytes/monocyte, B cell, and T cell lineages in individual mice are shown. HPCs = hematopoietic progenitor cells, HSCs = hematopoietic stem cells.

To analyze longer reconstitution, the PB of the surviving recipients after transplantation with 100 nHSC1/2 and nHPC1–4 cells but without competitor cells was retrospectively analyzed. As representatively shown in Figure 7, a significant level of myeloid reconstitution was detected in all surviving mice from 2 weeks after transplantation although its level was relatively low in the nHPC1 recipients. Interestingly, the myeloid-biased reconstitution pattern in which B and T lymphoid reconstitution gradually replaced myeloid reconstitution was observed for nHSC1 and nHPC2. The lymphoid-biased reconstitution pattern in which a high level of transient myeloid reconstitution was switched to lymphoid reconstitution 4 to 6 weeks after transplantation was observed for nHSC2. The common myeloid reconstitution pattern in which myeloid reconstitution was mainly reconstituted was observed for nHPC1. These data suggested that different types of HSC and HPC populations have radioprotection activity.

## 3 DISCUSSION

In the beginning of this study, we studied all cells among the KSL population (HSC1–3; HPC1–5) without gaps between its subpopulations (Fig. 1). Since they were continuous populations, their functional distinction among the populations appeared to be difficult. In the meantime, we also made a progress in the HSC purification and needed to update our protocol. We decided to reclassify the populations (nHSC1/2; nHPC1–4). The nHSC1 and nHPC1 populations were slightly purer than the HSC1 and HPC1 populations, respectively, and the nHSC2 population was significantly purer than the HSC3 population (Supplemental Fig. 1, Supplemental Table 1). The nHPC2, nHPC3, and nHPC4 populations, respectively, overlapped the MPP1/2, MPP3, and MPP4 (LMPP) populations (Supplemental Table 1).^19,20^ Of importance was that the old populations (HSC1–3; HPC1–5) covered all new populations (nHSC1/2; nHPC1–4) so that data from the old populations were interpretable. The nHSC1 population was enriched in myeloid-biased HSCs while the nHSC2 population was enriched in lymphoid-biased HSCs (Fig. 7).^14^ The HPC1/2 populations were enriched in myeloid-biased HPCs (Fig. 7).^14,18^

We have sometimes experienced early death of most lethally irradiated mice, even after co-transplantation with a sufficient number of competitor cells. Since we have never known the cause of early death, this has never been described in previously published papers. When we performed the CFU-S assay, lethally irradiated mice often died within 7 days after transplantation. We thus had to reduce an irradiation dose from 9.5 to 8.5 Gy in this study. Lethal dose may differ depending on the situations of specific pathogen-free animal facilities which are uncontrollable.

Radioprotection is a cell dose-dependent phenomenon.^1^ A large number of cells were required for radioprotection by megakaryocyte-erythrocyte progenitors.^5^ Only 100 nHSC2 cells were sufficient for 50% survival of lethally irradiated mice (Fig. 5). We could not determine how many nHSC1, nHPC1, and nHPC2 cells are required for 50% survival of lethally irradiated mice in this study. Nevertheless, we detected the radioprotection activity among the nHSC1/2 and nHPC1/2 populations, suggesting that radioprotection is specific to neither HSCs nor HPCs.

A relatively large proportion of CD48^+^ cells were excluded from the nHSC2 population. Accordingly, the proportion of nmEMk colonies in the total colonies formed by single nHSC2 cells increased (ZL and HE, unpublished data). The nmEMk differentiation potential was detected in the nHSC1/2 and nHPC1/2 populations which all exhibited radioprotection activity. In contrast, consistent with previous studies,^18,19^ the nHPC3/4 populations lacked the EMk differentiation potential, and did not exhibit the radioprotection activity. Since nHSC1 cells need more time to produce platelets and neutrophils after transplantation than do nHSC2 cells,^14^ the radioprotection activity in the nHSC1 population appeared to be lower than that in the nHSC2 population (Fig. 5).

Varying degrees of day 12 CFU-S activity was detected in the nHSC1/2 and nHPC1–4 populations although its activity was least in the nHPC4 population. Day 12 CFU-S activity was detected in the nHPC3 population. However, nHSC3 cells did not exhibit radioprotection activity. These data together suggested that the radioprotection activity is partially associated with the nmEMk differentiation potential and day 12 CFU-S activity.

Perhaps, all nHSC1/2 and nHPC1–4 cells are able to home to the bone marrow and spleen although their homing efficiencies may differ. It is interesting to know the localization of nHSC1/2 and nHPC1–4 cells under stress and physiological conditions. In particular, it is possible that the distribution of myeloid-biased HSCs differs from that of lymphoid-biased HSCs in the BM. If this is the case, their niches may also differ, and these 2 types of HSCs may be regulated in different manners.

Blood cells, particularly hematopoietic stem and progenitor cells are sensitive to ionizing radiation and undergo apoptosis when their DNA is severely damaged. Most mice die of infection and bleeding between 10 and 14 days after lethal irradiation. Reconstitution data (Figs. 6 and 7) together with previous data^14^ rather emphasized that lymphoid-biased HSCs and myeloid-biased HPCs could timely supply neutrophils and platelets and rescue lethally irradiated mice after these cells were transplanted. Perhaps, days 12–14 after irradiation would be the most critical time window for rescuing the mice. How neutrophils and platelets are rapidly and sufficiently produced by these cells but not the other cells remains to be clarified. Recently, we found that granulocyte colony-stimulating factor can directly stimulate the division of nHSC2 but not nHSC1 cells in vitro.^21^ It is possible that granulocyte colony-stimulating factor and thrombopoietin coordinately play a role in radioprotection by rapidly generating these blood cells from nHSC2 cells as well as nHPC1/2 cells.

## 4 MATERIALS AND METHODS

### 4.1 Mice

C57BL/6 (B6-CD45.2) mice were purchased from Beijing HFK Bioscience Co. (Beijing, China) and Sankyo Lab Service, Co. (Tokyo, Japan). B6-CD45.1 mice were bred and maintained at State Key Laboratory of Experimental Hematology. Only female mice were used. All animals were kept in certificated environment. The procedures for care and use of animals were approved by the Ethics Committee of Institute of Hematology & Blood Diseases Hospital, Chinese Academy of Medical Sciences, with approval number IHCAMS-DWLL-2017-KT001-1, date April-6, 2017. All applicable institutional and governmental regulations concerning the ethical use of animals were followed.

### 4.2 Isolation of HSCs and HPCs

For in vitro assays, bone marrow cells were collected from the femora, tibiae, and iliac crests of 8 to 10 week-old B6 mice and stained with antibodies. For flow cytometry sorting, c-Kit^+^Sca1^+^lineage^−^ (KSL) cells were divided into 8 populations as shown in Figure 1, based on previous studies.^13,14^

For in vivo assays, bone marrow cells were collected from B6-CD45.1 mice. c-Kit-positive cells were enriched and stained as described.^12^ KSL cells were divided into new HSC1–2, and HPC1–4 populations (nHSC1–2 and nHPC1–4, respectively). The relationships between the HSC1–3 and nHSC1,2 populations and between the HPC1–5 and the nHPC1–4 populations are demonstrated in Supplemental Fig. 1 and Supplemental Table 1.

### 4.3 Definition of HPCs

Unlike HSCs, HPCs have been poorly defined. In this study, we operationally defined HPCs as progenitors detected in the KSL population.

### 4.4 Single-cell colony assay

A 96-well U-bottom tissue culture plate (Falcon, Beckton Dickinson Labware, USA) in which each well had 200 μL of minimum essential medium (Sigma–Aldrich, USA) containing 10% fetal calf serum, 200 μM l-glutamine, 50 u/mL streptomycin/penicillin, 20 ng/mL recombinant murine stem cell factor, 20 ng/mL recombinant human thrombopoietin, 10 ng/mL murine interleukin-3, and 1 U/mL recombinant murine erythropoietin was prepared. Single cells were sorted to each well and cultured at 37°C and 5% CO_2_ in an Aztec air jacket incubator.

On days 7 and 14 of culture, the colony cells in each well were stained with antibodies. Flow-Count Fluorospheres (Beckman Coulter) were added to each well to estimate the cell number, and PI was added to exclude dead cells from analysis and sorting. Cells were then analyzed with a MACSQuant Analyzer (Miltineyi Biotec). The Gr1^+^CD115^−^, Gr1^−^CD115^+^, Gr1^−^CD115^−^CD41^+^, and Gr1^−^CD115^−^Ter119^+^ fractions were found to contain neutrophils (n), monocytes and macrophages (m), megakaryocytes (Mk), and erythroblasts (E), respectively. Cells in the CD41^+^Ter119^+^ fraction were operationally defined as progenitors (P). Cells with none of these lineage markers were shown as negative cells (Neg). Colony sizes were calculated using the following equation: P = C(T/B), where P is the population of the colony, C is the cell count from flow cytometric data, T is the total number of flow count fluorophores added to the sample, and B is the flow count fluorophore count from flow cytometric data. Colonies were defined as >200 live cells of any 1 lineage, as identified by flow cytometry. Clusters containing <200 live cells were excluded from the study.

### 4.5 Transplantation

nHSC1–2 cells; and nHPC1–4 cells isolated from B6-CD45.1 mice were transplanted via the tail vein into B6-CD45.2 mice irradiated at 8.5 Gy in split doses, 3 to 4 hours apart. After transplantation, the peripheral blood of the recipients was stained with antibodies, and analyzed with a FACSCanto using Summit software (Beckman Coulter) to detect CD45.1^+^ donor-derived Gr-1/Mac-1^+^ myeloid cells, B220^+^ B cells, and CD4/8^+^ T cells at the indicated time points.

### 4.6 Definition of radioprotection

If lethally mice transplanted with test donor cells survived >30 days after transplantation, test donor cells were considered to have radioprotection activity.

### 4.7 CFU-S assay and spleen fixation

The mice were sacrificed on days 7 and 12 after transplantation. Spleens were removed from the mice and fixed for 24 hours with Bouin solution made of 5 mL of 5% acetic acid, 75 mL of 0.9% picric acid, and 25 mL of 9% formaldehyde.

### 4.8 Statistical analysis

Student's two-tailed *t* test, ANOVA, and long-rank test were performed using Prism 6 software. Data were shown as mean ± SD.

## Supplementary Material

Supplemental Digital Content

## Supplementary Material

Supplemental Digital Content
