# Supplementary material for: Lymphoid-biased hematopoietic stem cells and myeloid-biased hematopoietic progenitor cells have radioprotection activity
Source: Blood Sci. 2021 Aug 19;3(4):113–21. doi: 10.1097/BS9.0000000000000089 (PMC8974907; doi:10.1097/BS9.0000000000000089)

**Supplemental figure legends**

**Supplemental Figure 1. Sorting gates for new HSC1, HSC2, HPC1, HPC2, HPC3, HPC4 populations**

The flow cytometry image shows the serial sorting gates. Lin^-^ cells were first gated on c-Kit-enriched cells. CD34-negative or CD34-positive cells were then gated on Lin^-^ cells. As shown in the upper panels, c-Kit^+^Sca-1^+^ (KS) cells were gated on CD34^-^Lin^-^ cells (CD34^-^KSL cells). CD34^-^KSL cells were fractionated into three populations based on their expression of CD41 and CD150. The nHSC1, nHSC2 and nHPC1 populations were finally sorted after the selection of CD201^+^CD48^-^ cells from each population. As shown in the middle panels, CD34^+^KSL cells were fractionated into the nHPC2, nHPC3 and nHPC4 populations based on the expression of Flt3 and CD150. Six populations were defined as follows: the nHSC1 (CD201^+^CD150^+^CD48^-^CD41^-^CD34^-^KSL), nHSC2 (CD201^+^CD150^-^CD48^-^CD41^-^CD34^-^KSL), nHPC1 (CD201^+^CD150^+^CD48^-^CD41^+^CD34^-^KSL), nHPC2 (CD150^+^Flt3^-^CD34^+^KSL), nHPC3 (CD150^-^Flt3^-^CD34^+^KSL), and nHPC4 (CD150^-^Flt3^+^CD34^+^KSL) populations.

The middle and lower panels show the relationship between HPC2-5 and nHPC2-4.


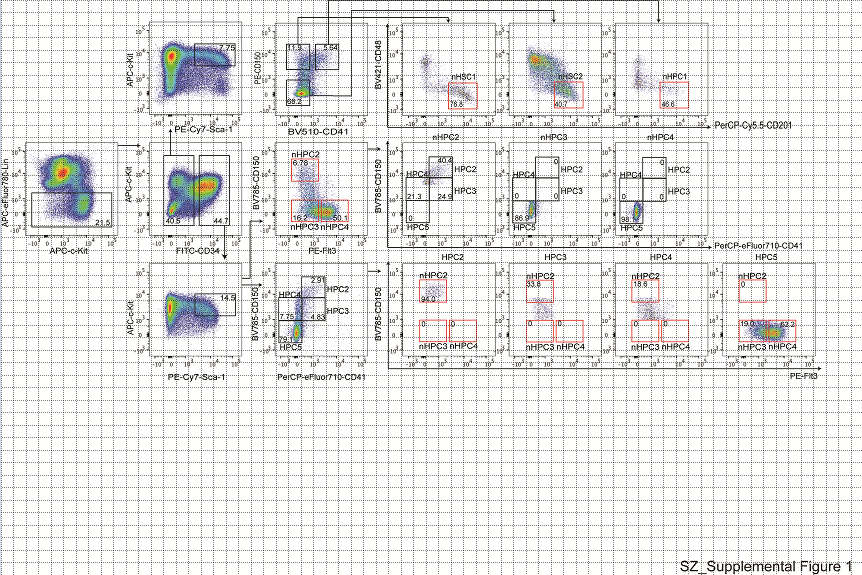

Supplement: Supplemental Digital Content [file bls-3-113-s001.docx]
