# Supplementary material for: Lymphoid-biased hematopoietic stem cells and myeloid-biased hematopoietic progenitor cells have radioprotection activity
Source: Blood Sci. 2021 Aug 19;3(4):113–21. doi: 10.1097/BS9.0000000000000089 (PMC8974907; doi:10.1097/BS9.0000000000000089)
Supplement: Supplemental Digital Content [file bls-3-113-s002.docx]

**Supplemental Table 1. The relationship among our old and new classifications of HSCs and HPCs, and Trumpp’s classification of HSCs and MPPs**

| This study | | Trumpp’s study |
| --- | --- | --- |
| New classification | Old classification |  |
| nHSC1 | HSC1 | HSC |
| CD201^+^CD48^-^CD150^+^CD41^-^  CD34^-^KSL cells | CD150^High^CD41^-^  CD34^-^KSL cells | CD150^+^CD48^-^  CD34^-^KSL cells |
| nHSC2 | HSC3 | (HSC-ST)* |
| CD201^+^CD48^-^ CD150^-^CD41^-^  CD34^-^KSL cells | CD150^Low^CD41^-^  CD34^-^KSL cells | CD150^-^CD48^-^  CD34^-^KSL cells |
| nHPC1 | HPC1 | - |
| CD201^+^CD48^-^CD150^+^CD41^+^  CD34^-^KSL cells | CD150^High^CD41^+^  CD34^-^KSL cells |  |
| nHPC2 | HPC2 | MPP1/2 |
| CD150^+^CD135^-^  CD34^+^KSL cells | CD150^High^CD41^+^  CD34^+^KSL cells | CD150^+^CD48^-/+^CD135^-^  CD34^+^KSL cells |
| nHPC3 | HPC5 | MPP3 |
| CD150^-^CD135^-^  CD34^+^KSL cells | CD150^Low^CD41^-^  CD34^+^KSL cells | CD150^-^CD48^+^CD135^-^  CD34^+^KSL cells |
| nHPC4 | HPC5 | MPP4 |
| CD150^-^CD135^+^  CD34^+^KSL cells | CD150^Low^CD41^-^  CD34^+^KSL cells | CD150^-^CD48^+^CD135^+^  CD34^+^KSL cells |

The corresponding cell populations are shown in rows.

CD34^-^c-Kit^+^Sca-1^+^lineage-negative (CD34^-^KSL) cells were separated into HSC1/2/3 and HPC1 populations (old classification) or nHSC1/2 and nHPC1 (new classification). CD34^+^KSL cells were separated into HPC2-5 (old classification), nHPC2-4 (new classification), or MPP1-4 populations (Trumpp’s classification). *, adapted population; -, no corresponding population. nHPC3 and 4 corresponded to MPP3 and 4, respectively, because most nHPC3 and 4 were positive for CD48 as shown in Supplemental Fig. S1 of our previously published paper (Xie M, et al., Haemetologica 2020). The nHSC1 population is enriched in myeloid-biased HSCs. The nHSC2 population is enriched in lymphoid-biased HSCs. The HPC1 population is enriched in common myeloid repopulating progenitors or theoretically CMP. The nHPC4 population is exactly the same as LMPP.
